# Mechanistic prediction of angelicae pubescentis radix in preventing arrhythmia via modulation of calcium channel targets: an integrated network pharmacology and target enrichment analysis

**DOI:** 10.3389/fphar.2026.1778679

**Published:** 2026-05-29

**Authors:** Duo Luo, Liang Hu

**Affiliations:** 1 Intensive Care Unit (ICU), The Third Affiliated Hospital (The First Hosptial of Nanchang), Jiangxi Medical College, Nanchang University, Nanchang, China; 2 Department of Traditional Chinese Medicine, The Third Affiliated Hospital (The First Hosptial of Nanchang), Jiangxi Medical College, Nanchang University, Nanchang, China

**Keywords:** angelicae pubescentis radix, arrhythmia, calcium signaling, cardioprotection, network pharmacology

## Abstract

**Objective:**

This study aimed to elucidate the potential mechanisms by which Angelicae Pubescentis Radix (APR) prevents arrhythmia through the regulation of calcium channels, based on network pharmacology, molecular docking, and *in vivo* validation.

**Methods:**

Active components of APR were screened from the TCMSP and PubChem databases under oral bioavailability (OB ≥ 30%) and drug-likeness (DL ≥ 0.18) criteria. Potential targets were predicted using SwissTargetPrediction and standardized using UniProt. Arrhythmia-related genes were collected from the GeneCards, OMIM, and TTD databases. Overlapping targets were identified for PPI network construction, GO/KEGG enrichment, and “component–target–pathway” network analysis. Core targets underwent molecular docking with key compounds. Furthermore, an isoproterenol (ISO)-induced arrhythmia rat model was established to evaluate the pharmacological effects of APR at different doses.

**Results:**

A total of potential 340 putative APR-related targets were identified from network pharmacology analysis. Among the APR-derived active compounds, three with the highest network connectivity (Angelol G, O-acetylcolumbianetin, and nodakenin) were selected for molecular docking. In parallel, 902 arrhythmia-related genes were retrieved, yielding 24 overlapping targets, among which five hub genes (PIK3CA, CREBBP, NR3C1, SCN10A, and KCNA5) were identified. KEGG analysis revealed significant enrichment in calcium signaling, adrenergic signaling, and PI3K-Akt/cAMP pathways. Molecular docking demonstrated strong binding affinities between Angelol G, O-acetylcolumbianetin, and nodakenin to NR3C1 and SCN10A. *In vivo*, APR reduced VT/VF incidence and duration, prolonged arrhythmia latency, improved LVEF and FS, and decreased serum levels of CK-MB, LDH, and cTnI. APR also decreased p-CaMKII (Thr286) and increased p-PLN (Ser16/Thr17) and SERCA2a, indicating improved calcium cycling.

**Conclusion:**

APR may exert anti-arrhythmic effects by targeting key regulatory nodes identified through network pharmacology, particularly PIK3CA, CREBBP, NR3C1, SCN10A, and KCNA5, and by exhibiting strong compound–target interactions in molecular docking. These network-predicted mechanisms were supported by *in vivo* findings, in which APR improved cardiac electrical stability, reduced myocardial injury, and restored Ca^2+^ handling through the CaMKII–PLN–SERCA2a axis. Collectively, these results highlight APR’s multi-target cardioprotective potential and provide mechanistic evidence for its application in arrhythmia prevention.

## Introduction

Cardiac arrhythmia is a prevalent and life-threatening cardiovascular disorder characterized by abnormal impulse generation or conduction, leading to irregular heart rhythms, hemodynamic instability, and an increased risk of sudden cardiac death (Kotadia et al., 2020). According to the Global Burden of Disease (GBD 2023) report, arrhythmias contribute to approximately 2.6 million deaths annually worldwide. Atrial fibrillation (AF) affects over 60 million people globally, and ventricular arrhythmias account for nearly 80% of sudden cardiac deaths. In China alone, epidemiological surveys indicate that the prevalence of AF exceeds 2.3% among adults over 40 years, and arrhythmia-related hospitalizations have increased by over 35% in the past decade, posing a growing challenge to healthcare systems (GBD 2023 Causes of Death Collaborators, 2025; Shi et al., 2022).

A substantial body of evidence indicates that dysregulation of intracellular Ca^2+^ homeostasis is a fundamental driver of arrhythmogenesis (Hamilton et al., 2021). Emerging studies suggest that several Ca^2+^-signaling related and electrophysiological regulatory targets, including PIK3CA, CREBBP, NR3C1, SCN10A, and KCNA5, play important roles in cardiomyocyte excitability and Ca^2+^ cycling. NR3C1 signaling has been linked to the transcriptional control of Ca^2+^ regulatory genes and redox balance, and its dysfunction can disturb sarcoplasmic reticulum Ca^2+^ turnover, creating a substrate for triggered arrhythmias (Cong et al., 2025; Lahiri et al., 2021). SCN10A, although classically regarded as a neuronal sodium channel, has been shown to influence cardiac excitability by altering late Na^+^ currents; this indirectly drives Ca^2+^ overload via Na^+^/Ca^2+^ exchanger activity, promoting delayed afterdepolarizations and malignant ventricular arrhythmias (Lahiri et al., 2021).

KCNA5, which governs the ultra-rapid delayed rectifier K^+^ current, also affects Ca^2+^ influx by shaping action potential duration; reduced KCNA5 activity prolongs depolarization and enhances Ca^2+^ entry, thus increasing susceptibility to atrial arrhythmias (Di et al., 2018). Meanwhile, PIK3CA-mediated PI3Kα signaling intersects with Ca^2+^ handling pathways to coordinate excitation–contraction coupling and structural remodeling, and its dysregulation promotes pro-arrhythmic Ca^2+^ cycling abnormalities (Huang et al., 2012). At the epigenetic level, CREBBP functions as a transcriptional co-activator that modulates chromatin accessibility for multiple ion-channel and Ca^2+^-handling genes; impaired CREBBP activity may contribute to pathological electrical remodeling that further destabilizes Ca^2+^ homeostasis (Sun et al., 2020).

Accumulating evidence also suggests that dysregulation of key Ca^2+^-handling proteins—particularly CaMKII, PLN, and SERCA2a—plays a central role in triggering delayed afterdepolarizations, abnormal Ca^2+^ cycling, and both atrial and ventricular arrhythmias (Luo and Anderson, 2013; Bers, 2002). Modulation of the CaMKII–PLN–SERCA2a axis has therefore emerged as a promising therapeutic approach for stabilizing intracellular Ca^2+^ homeostasis and reducing arrhythmic susceptibility.

Given this complex network of Ca^2+^-related targets, recent network pharmacology analyses suggest that Angelicae Pubescentis Radix (APR) may exert anti-arrhythmic effects by simultaneously modulating several of these key regulatory nodes. Such multi-target interactions offer a plausible mechanistic basis for stabilizing intracellular Ca^2+^ dynamics, reducing triggered activity, and ultimately preventing arrhythmia (Lu et al., 2020).

Current anti-arrhythmic drugs—such as β-blockers, sodium channel blockers, and calcium channel antagonists—have limited efficacy and potential proarrhythmic or cardiodepressive side effects, which restrict their long-term clinical use. Therefore, there is an urgent need to identify novel therapeutic agents that can modulate calcium-handling proteins in a more physiological and multi-targeted manner (Wang et al., 2023; Xiao et al., 2023).

In recent years, Traditional Chinese Medicine (TCM) has attracted increasing attention for its multi-component, multi-target, and pathway-network regulatory characteristics. Among these, APR, a commonly used herb in Chinese pharmacopoeia—has demonstrated anti-inflammatory, antioxidative, vasodilatory, and calcium channel–modulating effects (Lu et al., 2020). Pharmacological studies revealed that its main coumarin constituents (such as osthol, columbianadin, and imperatorin) can inhibit L-type Ca^2+^ currents, attenuate oxidative stress, and protect cardiomyocytes from ischemia/reperfusion injury (Xu et al., 2024; Yang et al., 2019). However, the specific molecular targets and signaling pathways through which APR exerts its anti-arrhythmic effects remain elusive.

Network pharmacology provides a systematic strategy to decode the complex interactions between multi-component herbal medicines and disease networks, enabling the prediction of key targets and signaling pathways. Combining this with molecular docking and experimental validation can reveal molecular-level interactions and establish credible pharmacological mechanisms (Noor et al., 2022; Zhai et al., 2025).

In this study, we integrated network pharmacology, target enrichment analysis, molecular docking, and *in vivo* experiments to elucidate the anti-arrhythmic mechanism of APR. We hypothesized that APR exerts its cardioprotective effect by modulating calcium regulatory and signaling associated targets (PIK3CA, CREBBP, NR3C1, SCN10A, and KCNA5) and restoring Ca^2+^ homeostasis. Given that dysregulation of key Ca^2+^-handling proteins—particularly CaMKII, phospholamban (PLN), and SERCA2a—is known to promote sarcoplasmic reticulum Ca^2+^ leak, delayed afterdepolarizations, and ventricular arrhythmias, APR may also confer protection by influencing this regulatory axis. This work aims to bridge the gap between traditional herbal pharmacology and modern molecular cardiology, providing theoretical and experimental evidence for the application of APR in arrhythmia prevention and treatment.

## Materials and methods

### Collection and screening of active compounds in APR

The chemical constituents of APR were collected from the Traditional Chinese Medicine Systems Pharmacology Database (TCMSP) (https://tcmsp-e.com/) and PubChem (https://pubmed.ncbi.nlm.nih.gov/). Compounds were filtered based on oral bioavailability (OB) ≥ 30% and drug-likeness (DL) ≥ 0.18, which are commonly used screening thresholds in TCMSP-based network pharmacology studies to identify compounds with relatively favorable absorption characteristics and drug-like properties. These criteria were applied to improve the likelihood of selecting bioactive compounds with potential *in vivo* pharmacological relevance (Noor et al., 2022; Zhai et al., 2025). The structures and SMILES formats of eligible compounds were obtained for further analysis. After eliminating duplicates, a set of OB/DL-qualified compounds was obtained. Among these, three compounds with the highest topological connectivity in the compound–target network—Angelol G, O-acetylcolumbianetin, and nodakenin—were selected as the representative active components of APR for subsequent target prediction and molecular docking analyses.

### Prediction of potential targets and arrhythmia-related genes

The potential targets of the selected compounds were predicted using the SwissTargetPrediction (http://www.swisstargetprediction.ch/) and TCMSP databases (species: *Homo sapiens*). Predicted targets were standardized using the UniProt database (https://www.uniprot.org/) for consistent gene nomenclature.

Arrhythmia-related genes were retrieved from GeneCards (https://www.genecards.org/), OMIM (https://omim.org/), and the Therapeutic Target Database (TTD) (http://db.idrblab.net/ttd/), using the search terms “arrhythmia,” “atrial fibrillation,” and “ventricular tachycardia.” Duplicates were removed and the intersecting targets between APR and arrhythmia were identified using a Venn diagram to obtain potential anti-arrhythmic targets.

### Network construction and enrichment analysis

The intersecting targets were imported into the STRING database (https://string-db.org/) for protein–protein interaction (PPI) analysis with a confidence score threshold of 0.7. The resulting interaction network was visualized using Cytoscape (v3.9.1), and hub genes were identified using the Degree and Maximal Clique Centrality (MCC) algorithms in the CytoHubba plug-in. Functional enrichment analysis was conducted using DAVID 6.8 (https://david.ncifcrf.gov/), including Gene Ontology (GO) annotation and Kyoto Encyclopedia of Genes and Genomes (KEGG) pathway enrichment with P < 0.05 considered statistically significant. Results were visualized as bubble plots highlighting biological processes and pathways associated with calcium signaling, adrenergic signaling, and PI3K–Akt signaling.

### Molecular docking analysis

To verify the predicted compound–target interactions, molecular docking was performed between the top five hub proteins (PIK3CA, CREBBP, NR3C1, SCN10A, and KCNA5) and representative active compounds (Angelol G, O-acetylcolumbianetin, and nodakenin). Default parameters were used in AutoDock Vina unless otherwise specified, and the conformation with the lowest binding energy was selected as the optimal docking result.

### Protein and ligand preparation

The 3D structures of the target proteins were obtained from the Protein Data Bank (PDB), and ligand structures were optimized using Chem3D. Both proteins and ligands were converted into PDBQT format using AutoDockTools (v1.5.7).

### Docking procedure and visualization

Docking was performed using AutoDock Vina (v1.1.2). The docking grid box was centered around the known active site of each protein. Binding energies < −7.0 kcal/mol were considered indicative of strong binding affinity, which is a commonly used threshold in molecular docking studies to identify compounds with potential biological relevance, particularly in the absence of experimentally validated control ligands or known binders. The docking conformations were visualized using PyMOL (v2.5) and Discovery Studio Visualizer, and a compound–target–pathway interaction network was constructed using Cytoscape.

### Experimental animals and grouping

Male Sprague–Dawley (SD) rats (200 ± 20 g) were obtained from an accredited laboratory animal center and acclimatized for 7 days under controlled environmental conditions (22 °C ± 2 °C, 55% ± 10% humidity, 12 h light/dark cycle). All procedures were conducted in accordance with the National Institutes of Health Guide for the Care and Use of Laboratory Animals and approved by the Animal Ethics Committee of Guangdong Provincial Laboratory Animal Center (Approval No. D202505-18).

Rats were randomly assigned to four groups (n = 6 per group): vehicle, ISO, APR-L, and APR-H. Randomization was performed using a random number table. To explore the protective effect of APR on arrhythmia, we established a rat arrhythmia model using isoproterenol hydrochloride (ISO) (CAS No. 6078–56–4, Sigma-Aldrich). Rats received subcutaneous injections of ISO (5 mg/kg/day) for two consecutive days to induce myocardial stress and electrical instability. APR extract, prepared as described below, was administered orally once daily for 7 days prior to ISO induction. Two doses were selected—50 mg/kg (low dose) and 200 mg/kg (high dose)—based on preliminary pharmacological screening. Control rats received the same volume of 0.5% carboxymethyl cellulose sodium (CMC-Na) by gavage, while the ISO group received vehicle pretreatment without APR. All outcome assessments, including arrhythmia evaluation and echocardiographic measurements, were conducted by investigators blinded to group allocation.

### Preparation of APR extract

Dried roots of APR were pulverized and extracted with 80% ethanol (solid–liquid ratio 1:12, w/v) under ultrasonic-assisted extraction (30 °C, 240 W, 20 min). The filtrate was concentrated and suspended in 0.5% CMC-Na for oral administration.

### Evaluation of Arrhythmia Latency and Cardiac function.

Following ISO administration, the onset latency and duration of ventricular premature beats (VP) and ventricular tachycardia (VT) were continuously observed for 60 min using a physiological monitoring system. Arrhythmia severity was graded on a five-point scale (0–5) according to established ISO-induced arrhythmia criteria.

Cardiac function was assessed using a high-frequency ultrasound imaging system (Mindray M9, probe model L20-5s, Cat. No. 065-000525-00, Shenzhen Mindray Bio-Medical Electronics Co., Ltd., China). The following parameters were measured: left ventricular ejection fraction (LVEF), fractional shortening (FS), heart rate (HR), and E/A ratio (early-to-late diastolic filling ratio). All analyses were performed by a blinded observer.

### Determination of serum myocardial injury markers

Blood samples were collected from the abdominal aorta 24 h after the last ISO injection. Serum was separated by centrifugation (3,000 rpm, 10 min, 4 °C). Levels of creatine kinase-MB (CK-MB), lactate dehydrogenase (LDH), and cardiac troponin I (cTnI) were measured using ELISA kits (Nanjing Jiancheng Bioengineering Institute, China) following the manufacturer’s instructions. Absorbance was measured at 450 nm with a microplate reader (BioTek, United States).

### Measurement of myocardial Ca^2+^ content

Left ventricular tissue was homogenized in ice-cold physiological buffer. Intracellular Ca^2+^ concentration was determined using a Calcium Assay Kit (Cat. No. C004-2, Nanjing Jiancheng Bioengineering Institute, Nanjing, China). According to the manufacturer’s instructions. Results were expressed as nmol/mg of total protein.

### Western blot analysis

Protein was extracted from left ventricular tissue using RIPA lysis buffer containing protease and phosphatase inhibitors. Equal amounts of protein (30 μg) were separated on 10% SDS–PAGE gels and transferred onto PVDF membranes. After blocking with 5% nonfat milk for 1 h, membranes were incubated overnight at 4 °C with the following primary antibodies: p-CaMKII (Thr286) (ab124880, Abcam),p-PLN (Ser16) (ab15000, Abcam),p-PLN (Thr17) (AP0910, Sigma),SERCA2a (ab137020, Abcam) and GAPDH (ab9485, Abcam). After washing, membranes were incubated with HRP-conjugated secondary antibodies (ab6721, Abcam) for 1 h at room temperature. Protein bands were visualized using enhanced chemiluminescence (ECL, Thermo Fisher) and quantified using ImageJ software (NIH, United States). Protein expression was normalized to GAPDH.

### Statistical analysis

All experiments were independently repeated at least three times. Data were expressed as mean ± standard deviation (SD). Statistical analyses were performed using IBM SPSS Statistics, version 23.0 (IBM Corp., Armonk, NY, United States). Data normality was assessed using the Shapiro–Wilk test. Comparisons between two groups were analyzed using the unpaired Student’s t-test, while comparisons among multiple groups were performed using one-way ANOVA followed by Tukey’s *post hoc* test. A difference of P < 0.05 was considered statistically significant.

## Results

### Systematic framework integrating network pharmacology, molecular docking, and experimental validation

**FIGURE 1:**
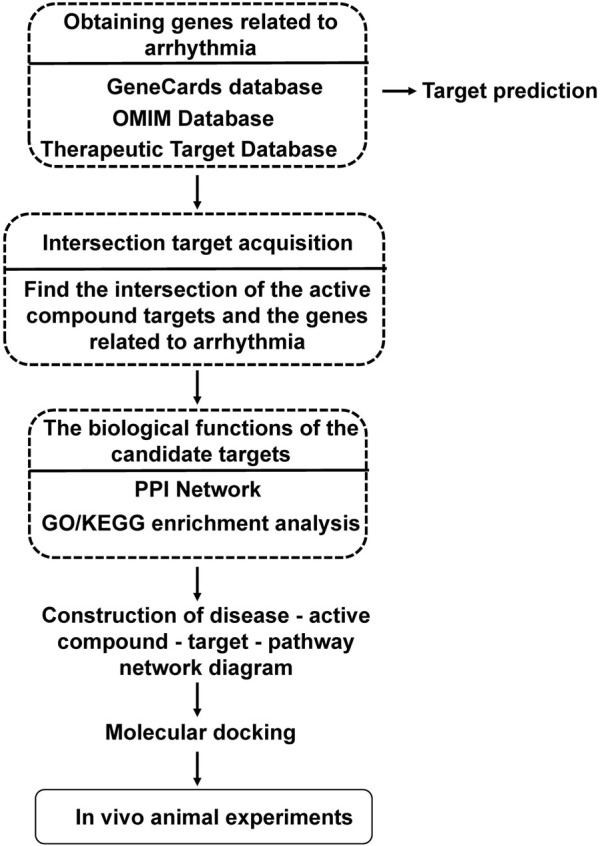
Workflow of the study based on network pharmacology and experimental validation.

To elucidate the potential mechanisms underlying the anti-arrhythmic effects of APR, network pharmacology analysis was performed to integrate drug–target–disease interactions. The overall workflow of the study is illustrated in Figure 1, which includes target prediction, intersection analysis, functional enrichment, network construction, molecular docking, and *in vivo* experimental validation.

### Network pharmacology-based identification of potential anti-arrhythmic targets of APR

**FIGURE 2:**
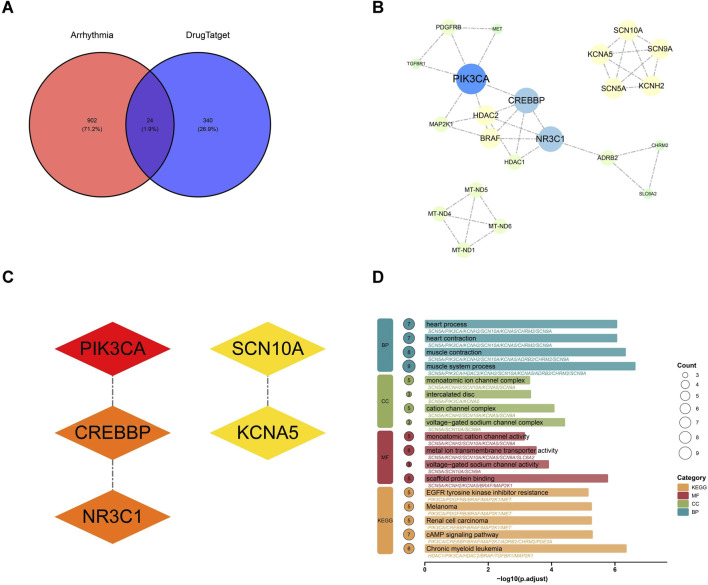
Network pharmacology analysis of APR against arrhythmia. **(A)** Venn diagram showing the overlapping targets between arrhythmia-related genes and predicted drug targets of APR. **(B)** Protein–protein interaction (PPI) network of the intersecting targets constructed using the STRING database. **(C)** Visualization of hub gene relationships within the network. **(D)** GO and KEGG enrichment analysis of the common targets, illustrating enriched biological processes, molecular functions, cellular components, and pathways.

A total of 340 putative targets of APR were identified from TCMSP and SwissTargetPrediction databases, while 902 arrhythmia-related genes were retrieved from GeneCards, OMIM, and TTD. The intersection analysis yielded 24 shared targets, accounting for 1.9% of all identified genes, which were considered the candidate anti-arrhythmic targets of APR (Figure 2A).

Subsequent protein–protein interaction (PPI) network analysis revealed that these intersection targets formed a highly connected network, with PIK3CA, CREBBP, NR3C1, SCN10A, and KCNA5 emerging as hub nodes (Figure 2B). Among them, PIK3CA and CREBBP were positioned at the core of the interaction network, regulating intracellular signal transduction and transcriptional modulation, whereas SCN10A and KCNA5, key sodium and potassium channel subunits, were enriched in ion conduction modules. These findings suggested that APR exerts its pharmacological effects through simultaneous modulation of signaling transduction and ion channel regulation, potentially restoring cardiac electrical stability.

To further clarify the relationship among hub genes, a subnetwork was constructed to visualize their topological connections (Figure 2C). The PIK3CA–CREBBP–NR3C1 axis represented the transcriptional and signaling core, whereas SCN10A and KCNA5 formed a complementary ion channel regulatory branch. This pattern indicates that APR may act as a dual-modulator of intracellular kinase signaling and membrane electrophysiological homeostasis.

Gene Ontology (GO) and KEGG pathway enrichment analyses were subsequently performed to explore the biological significance of these targets (Figure 2D). The biological process (BP) terms were mainly enriched in heart contraction, muscle system process, and regulation of cardiac rhythm. The cellular component (CC) terms focused on voltage-gated ion channel complexes and intercalated discs, while molecular function (MF) analysis revealed significant enrichment in cation transmembrane transporter activity and calcium channel binding. KEGG analysis further demonstrated that the common targets were primarily involved in calcium signaling pathway, cAMP signaling pathway, PI3K-Akt signaling pathway, and adrenergic signaling in cardiomyocytes—all crucial for excitation–contraction coupling and arrhythmia regulation.

### Molecular docking validation of the binding between key active compounds and core targets

To further verify the binding activity between APR-derived compounds and core targets involved in calcium regulation, molecular docking was performed. The top five hub proteins (PIK3CA, CREBBP, NR3C1, SCN10A, and KCNA5) were docked with the top three active components. Among these targets, NR3C1 and SCN10A demonstrated the most favorable and stable interactions with APR constituents, and thus were selected for detailed structural visualization and analysis.

**FIGURE 3:**
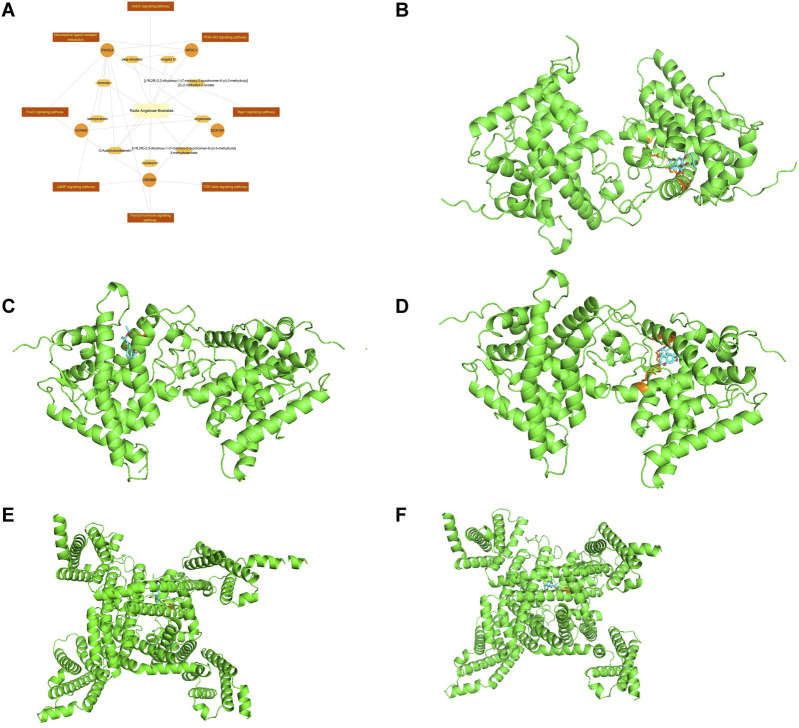
Molecular docking analysis of APR-derived compounds with core targets associated with Ca^2+^-related signaling. **(A)** Compound–target–pathway network showing the association among APR bioactive compounds, core targets, and enriched signaling pathways. **(B–F)** Docking models displaying the 3D binding conformations between representative active compounds and their respective targets: **(B)** [(1R, 2R)-2,3-dihydroxy-1-(7-methoxy-2-oxochromen-6-yl)-3-methylbutyl] (Z)-2-methylbut-2-enoate–NR3C1, **(C)** O-acetylcolumbianetin–NR3C1, **(D)** nodakenin–NR3C1, **(E)** O-acetylcolumbianetin–SCN10A, and **(F)** [(1R, 2R)-2,3-dihydroxy-1-(7-methoxy-2-oxochromen-6-yl)-3-methylbutyl] (Z)-2-methylbut–SCN10A. The protein structures are shown in green ribbon format, and ligands are represented as colored sticks.

As shown in Figure 3A, the compound–target–pathway network highlighted strong associations between APR bioactive components and the key regulatory genes NR3C1 and SCN10A. NR3C1 acts as an upstream transcriptional regulator that can indirectly affect Ca^2+^-handling pathways, whereas SCN10A modulates cardiac excitability and influences Ca^2+^ dynamics through Na^+^–Ca^2+^ coupling. These findings suggest that both nodes may contribute to APR’s putative anti-arrhythmic activity. Table 1 summarizes the docking scores for the five representative ligand–target complexes visualized in Figures 3B–F, each corresponding to one of the lowest binding-energy conformations identified. Among all ligand–target interactions, [(1R,2R)-2,3-dihydroxy-1-(7-methoxy-2-oxochromen-6-yl)-3-methylbutyl] (Z)-2-methylbut-2-enoate demonstrated the highest predicted affinity for NR3C1, with a docking score of −10.3 kcal/mol (Figure 3B). The compound formed stable hydrogen bonds and hydrophobic contacts within the glucocorticoid receptor binding cavity, indicating a high degree of structural complementarity.

**TABLE 1:** Molecular docking results of APR compounds with key predicted targets.

Target	ID	MOL-ID	Compound	DockingScore
NR3C1	1M2Z	MOL004778	[(1R,2R)-2,3-dihydroxy-1-(7-methoxy-2-oxochromen-6-yl)-3-methylbutyl] (Z)-2-methylbut-2-enoate	−10.3
NR3C1	1M2Z	MOL003608	O-acetylcolumbianetin	−9.4
NR3C1	1M2Z	MOL004792	nodakenin	−9.4
SCN10A	7WE4	MOL003608	O-acetylcolumbianetin	−8.6
SCN10A	7WE4	MOL004778	[(1R,2R)-2,3-dihydroxy-1-(7-methoxy-2-oxochromen-6-yl)-3-methylbutyl] (Z)-2-methylbut-2-enoate	−8.6

Similarly, O-Acetylcolumbianetin and nodakenin also showed strong binding to NR3C1, each yielding docking scores of −9.4 kcal/mol (Figures 3C,D). These ligands interacted with residues located in regulatory regions of the receptor, suggesting their potential to influence NR3C1- associated signaling processes relevant to Ca^2+^ handling.

In addition, SCN10A demonstrated stable interactions with the two ligands shown in Figures 3E,F (MOL003608 and MOL004778), both exhibiting docking scores of −8.6 kcal/mol. The compounds occupied pockets near the transmembrane domain, forming hydrogen bonds and hydrophobic interactions that may affect sodium-dependent excitability and indirectly modulate intracellular Ca^2+^ loading.

### APR restores cardiac function and reduces arrhythmia susceptibility in ISO-induced rats.

**FIGURE 4:**
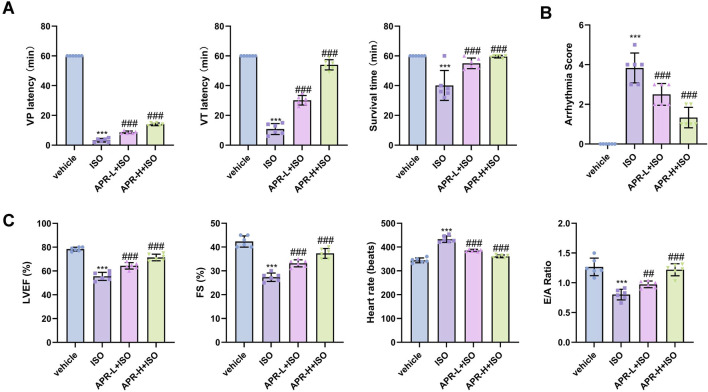
Effects of APR on arrhythmic susceptibility and cardiac function in ISO-induced rats. **(A)** APR pretreatment prolonged ventricular premature (VP) and ventricular tachycardia (VT) latency and increased survival time in a dose-dependent manner. Animals without arrhythmic events were assigned a latency of 60 min (observation endpoint). **(B)** Quantification of arrhythmia scores showing reduced arrhythmic severity following APR treatment. **(C)** Echocardiographic parameters showing APR improved left ventricular ejection fraction (LVEF), fractional shortening (FS), reduced heart rate, and restored the E/A ratio compared with ISO. n = 6 per group. Statistical analysis was performed using one-way ANOVA followed by Tukey’s *post hoc* test. ***P < 0.001 vs. vehicle; ##P < 0.01, ###P < 0.001 vs. ISO.

To validate the network pharmacology predictions, the protective effect of APR was evaluated in an ISO-induced arrhythmia rat model. Compared with the vehicle group, ISO administration markedly shortened the ventricular premature (VP) and ventricular tachycardia (VT) latency, and significantly reduced the survival time after arrhythmia onset (P < 0.001). Pretreatment with APR notably prolonged both VP and VT latency and increased survival time in a dose-dependent manner (P < 0.001 vs. ISO), indicating that APR enhanced resistance to arrhythmic events (Figure 4A).

In addition, quantitative analysis of arrhythmia scores demonstrated a substantial increase in the ISO group compared with the control (P < 0.001), whereas APR pretreatment significantly reduced arrhythmia severity in a dose-dependent manner (P < 0.001 vs. ISO), further confirming its cardioprotective effect (Figure 4B).

Echocardiographic analysis revealed that ISO-induced cardiac dysfunction was characterized by decreased left ventricular ejection fraction (LVEF) and fractional shortening (FS), accompanied by increased heart rate and a reduced E/A ratio, suggesting impaired diastolic relaxation. APR administration effectively reversed these abnormalities. Both low- and high-dose APR significantly increased LVEF and FS, reduced heart rate, and restored the E/A ratio compared with ISO-treated rats (P < 0.001; for E/A ratio in the APR-L group, P = 0.0068), with the high-dose group showing near-complete functional recovery (Figure 4C).

### APR reduces myocardial injury biomarkers in ISO-induced arrhythmia rats

**FIGURE 5:**
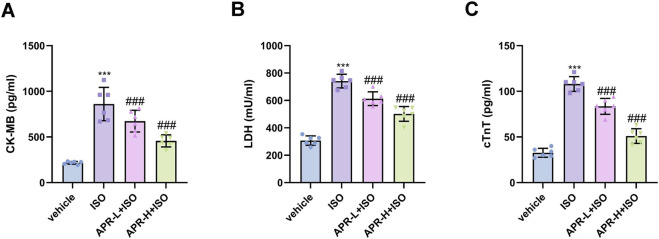
Effects of APR on serum myocardial injury biomarkers in ISO-induced rats. **(A)** Serum creatine kinase-MB (CK-MB) levels. **(B)** Serum lactate dehydrogenase (LDH) levels. **(C)** Serum cardiac troponin I (cTnI) levels. N = 6 per group. Statistical analysis was performed using one-way ANOVA followed by Tukey’s *post hoc* test. ***P < 0.001 vs. vehicle; ###P < 0.001 vs. ISO.

To further evaluate the cardioprotective effect of APR *in vivo*, serum levels of myocardial injury markers were measured. As shown in Figures 5A–C, ISO administration significantly increased serum levels of creatine kinase-MB (CK-MB; Figure 5A), lactate dehydrogenase (LDH; Figure 5B), and cardiac troponin I (cTnI; Figure 5C) compared with the vehicle group (P < 0.001), indicating severe myocardial injury and membrane disruption.

Pretreatment with APR markedly attenuated these elevations in a dose-dependent manner. Both low- and high-dose APR significantly reduced serum CK-MB, LDH, and cTnI levels compared with ISO alone (P < 0.001), with the high-dose group showing the most pronounced protective effect. These findings demonstrate that APR effectively mitigates ISO-induced myocardial damage, reflecting its capacity to preserve cardiomyocyte integrity and reduce ischemic injury associated with arrhythmia.

### APR restores calcium homeostasis by regulating the CaMKII–PLN–SERCA2a axis in ISO-induced rats

**FIGURE 6:**
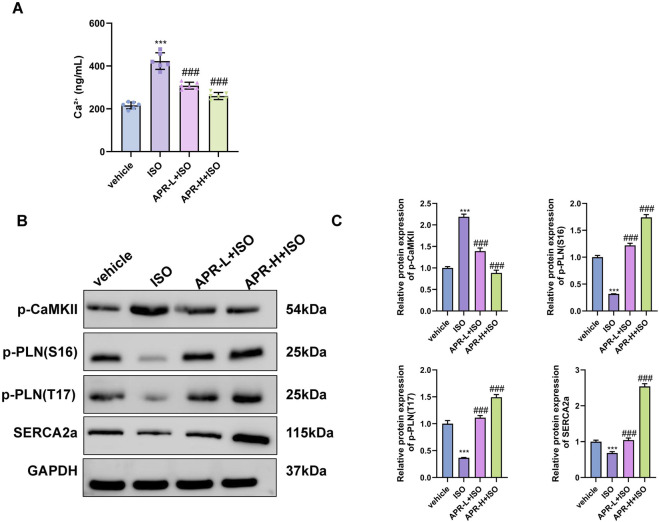
Effects of APR on myocardial Ca^2+^ levels and the CaMKII–PLN–SERCA2a signaling pathway in ISO-induced rats. **(A)** Myocardial Ca^2+^ concentration measured using a colorimetric assay. n = 6 per group. **(B)** Representative Western blot images showing p-CaMKII (Thr286), p-PLN (Ser16), p-PLN (Thr17), SERCA2a, and GAPDH expression in each group. **(C)** Quantitative analysis of relative protein expression levels of p-CaMKII, p-PLN (Ser16), p-PLN (Thr17), and SERCA2a. n = 3 per group. Statistical analysis was performed using one-way ANOVA followed by Tukey’s *post hoc* test. ***P < 0.001 vs. vehicle; ###P < 0.001 vs. ISO.

To further confirm whether APR regulates calcium-handling mechanisms during arrhythmia, myocardial calcium content and the expression of calcium regulatory proteins were assessed. As shown in Figure 6A, ISO administration led to a significant elevation in myocardial Ca^2+^ concentration compared with the vehicle group (P < 0.001), indicating marked calcium overload. Pretreatment with APR notably reduced myocardial Ca^2+^ accumulation in a dose-dependent manner (P < 0.001 vs. ISO), suggesting that APR alleviates ISO-induced calcium dysregulation.

Western blot analysis demonstrated significant alterations in the CaMKII–PLN–SERCA2a signaling axis following ISO treatment. Specifically, ISO markedly upregulated phosphorylated CaMKII (p-CaMKII, Thr286) and downregulated phosphorylated PLN (p-PLN, Ser16 and Thr17) and SERCA2a expression compared with the vehicle group (P < 0.001). APR pretreatment effectively reversed these changes in a dose-dependent manner, as shown in Figures 6B,C. Both low- and high-dose APR significantly suppressed p-CaMKII overactivation and enhanced p-PLN and SERCA2a expression levels (P < 0.001 vs. ISO). The high-dose APR group exhibited near-complete restoration of the CaMKII–PLN–SERCA2a axis, consistent with improved calcium handling and reduced arrhythmia susceptibility.

## Discussion

This study combined network pharmacology, molecular docking, and *in vivo* validation to elucidate how APR protects against arrhythmia through calcium-regulatory and electrophysiological mechanisms. The main findings demonstrate that APR exerts significant anti-arrhythmic and cardioprotective effects by modulating the five core hub genes—PIK3CA, CREBBP, NR3C1, SCN10A, and KCNA5—identified through network topology analysis. Molecular docking suggested strong interactions between network-selected APR coumarins and the glucocorticoid receptor NR3C1 and the sodium channel SCN10A, whereas *in vivo* studies showed that APR effectively reduced arrhythmia susceptibility, alleviated myocardial injury, and improved Ca^2+^ handling. However, based on the current data, it remains difficult to determine whether APR directly activates or inhibits SCN10A and NR3C1 activity. The molecular docking results primarily indicate potential binding interactions rather than functional modulation, and the directionality of these effects requires further experimental validation. Moreover, although the *in silico* predictions and *in vivo* findings are generally consistent, the mechanistic link between compound–target interactions and physiological outcomes remains associative. Additional studies are needed to establish a direct causal relationship between APR-mediated target modulation and its anti-arrhythmic effects. Together, these results suggest that APR may modulate transcriptional regulators, ion-channel related proteins, and Ca^2+^-handling pathways that converge to stabilize cardiac electrical activity.

The *in vivo* validation further supported these bioinformatic predictions. ISO-induced arrhythmic rats exhibited characteristic features of calcium overload, electrical instability, and myocardial injury, all of which were significantly attenuated by APR (Gupta et al., 2024). APR treatment prolonged arrhythmic latency, improved cardiac contractility, and reduced serum myocardial enzyme leakage, confirming that the network-derived targets translate into measurable physiological benefits (Wu et al., 2018). Thus, the integrative approach delineated a coherent mechanistic pathway linking APR’s molecular interactions to functional protection.

Disruption of intracellular calcium homeostasis is widely recognized as a central driver of arrhythmogenesis. Under pathological stimulation, excessive cytosolic Ca^2+^ accumulation activates downstream calcium-responsive kinases and transcriptional regulators. This ultimately disturbs the coordinated processes of calcium uptake, release, and extrusion. Such imbalance suppresses the phosphorylation of phospholamban (PLN) at Ser16 and Thr17, reduces SERCA2a-mediated Ca^2+^ reuptake into the sarcoplasmic reticulum (SR), and promotes diastolic Ca^2+^ leak and spontaneous SR Ca^2+^ release events. These alterations form the substrate for delayed afterdepolarizations, conduction instability, and increased vulnerability to ventricular tachyarrhythmias (Ruiz-Hurtado et al., 2012; Edwards and Blatter, 2014).

Accumulating evidence suggests that this maladaptive Ca^2+^ cycling is influenced not only by membrane ion channels but also by upstream regulatory genes involved in stress response, transcriptional control, and intracellular signaling. In this context, the calcium-associated hub genes identified in our network—PIK3CA, CREBBP, NR3C1, SCN10A, and KCNA5—have been implicated in modulating cardiac excitability, ion flux balance, and kinase-dependent Ca^2+^ handling pathways. Thus, improving the regulatory balance governed by these targets may offer a multifaceted means to stabilize intracellular Ca^2+^ dynamics and prevent arrhythmia development (Landstrom et al., 2017).

Our findings demonstrate that APR effectively interrupts this pathological cascade. By suppressing CaMKII hyperactivation and restoring PLN phosphorylation (Ser16 and Thr17), APR relieves the inhibitory effect of PLN on SERCA2a, thereby promoting SR Ca^2+^ reuptake and normalizing intracellular Ca^2+^ cycling. These findings are consistent with previous studies demonstrating that CaMKII-dependent regulation of phospholamban plays a central role in cardiac Ca^2+^ handling and arrhythmogenesis. In particular, CaMKII-mediated phosphorylation of PLN has been shown to critically modulate SERCA2a activity and sarcoplasmic reticulum Ca^2+^ cycling in cardiac disease, as comprehensively reviewed by Mattiazzi and Kranias (Mattiazzi and Kranias, 2014). Thus, the observed changes in p-CaMKII and p-PLN in this study align with this established regulatory framework and further support the involvement of this axis in the anti-arrhythmic effects of APR. This reestablishment of calcium balance aligns with the observed improvements in left ventricular function and the reduced arrhythmic burden. To further substantiate the role of the CaMKII–PLN–SERCA2a axis, future studies could incorporate pharmacological modulation of SERCA2a activity. For example, the use of SERCA inhibitors such as thapsigargin (TG) or cyclopiazonic acid (CPA), as well as SERCA activators (e.g., CDN1163), may help determine whether altering sarcoplasmic reticulum Ca^2+^ uptake directly influences the anti-arrhythmic effects of APR. Such approaches would provide more definitive evidence for the causal involvement of this pathway. In summary, APR acts as a functional modulator of Ca^2+^ homeostasis, targeting both upstream calcium influx and downstream reuptake regulation.

Previous studies have shown that synthetic CaMKII inhibitors and SERCA2a activators can prevent arrhythmia by stabilizing Ca^2+^ handling (Anderson, 2021), but their clinical use is limited by narrow therapeutic windows and adverse cardiac effects. In contrast, APR achieves similar regulation via multi-target coordination of natural compounds, offering a potentially safer therapeutic strategy.

Coumarins—such as osthol and columbianadin—are well-documented for their calcium antagonistic, antioxidant, and anti-inflammatory properties (Su et al., 2019). Recent reports have indicated that osthol suppresses L-type calcium currents and reduces ventricular arrhythmia in ischemic injury models (Zhang et al., 2023). Our findings extend these observations by revealing a broader, system-level mechanism: APR not only inhibits Ca^2+^ influx but also regulates CaMKII-mediated phosphorylation cascades, positioning it as a network-level modulator of calcium homeostasis. This dual action distinguishes APR from conventional single-target calcium blockers, emphasizing the integrative pharmacology inherent to traditional herbal medicine.

The therapeutic advantage of APR lies in its ability to act on multiple nodes within the calcium regulatory network. By simultaneously influencing CaMKII, PLN, and SERCA2a, APR exerts a coordinated effect that stabilizes electrical conduction, reduces cellular Ca^2+^ overload, and preserves contractile function (Lu et al., 2020). Such multi-target synergy aligns with the core philosophy of Traditional Chinese Medicine (TCM), which emphasizes restoring systemic balance rather than blocking a single pathological pathway (Zhao et al., 2023).

Moreover, the enrichment of APR targets in PI3K–Akt and cAMP signaling pathways suggests that its cardioprotective effects may extend beyond calcium regulation to include anti-apoptotic and metabolic regulatory functions, consistent with the holistic nature of herbal therapeutics. This systems-level regulation provides a theoretical framework for understanding how complex natural compounds achieve integrative cardiovascular protection.

While the current study establishes a strong mechanistic foundation, several limitations warrant attention. First, the molecular docking predictions require confirmation through biophysical interaction assays (e.g., surface plasmon resonance or thermal shift analysis). As molecular docking is inherently predictive, it does not provide direct evidence of target engagement, and further biochemical validation is required to confirm the interactions between APR-derived compounds and the identified targets. In addition, due to the limited availability of experimentally validated control ligands or known binders for certain targets, no reference compounds were included for direct comparison, which may limit the quantitative interpretation of binding affinities. Second, beyond molecular interaction validation, electrophysiological validation using patch-clamp techniques is essential to quantify the direct effects of APR on calcium and potassium currents (Che and Zhang, 2025). Third, this work employed an acute arrhythmia model; future studies should include chronic models and human-induced pluripotent stem cell–derived cardiomyocytes to assess translational relevance. Furthermore, the contribution of other active components within APR beyond coumarins should be explored using multi-omics approaches (proteomics, phosphoproteomics, metabolomics) to map the complete regulatory landscape. Integrating these datasets with computational modeling of calcium dynamics may reveal additional synergistic interactions and therapeutic targets. Moreover, due to the multi-component nature of APR, potential off-target effects and interactions among different bioactive constituents cannot be excluded, which may extend beyond the pathways identified in the present study and contribute to the overall pharmacological complexity.

## Conclusion

In conclusion, this study provides comprehensive evidence that APR prevents arrhythmia through restoration of intracellular calcium homeostasis and regulation of the CaMKII–PLN–SERCA2a signaling axis. By acting on multiple targets within the calcium-handling network, APR alleviates Ca^2+^ overload, stabilizes myocardial excitation–contraction coupling, and protects against electrical remodeling. These findings not only clarify the molecular basis of APR’s anti-arrhythmic effects but also exemplify how modern integrative pharmacology can decode the multi-component, multi-target mechanisms of traditional herbal medicine, bridging ancient therapeutics with contemporary cardiovascular science.

## Data Availability

The datasets presented in this study can be found in online repositories. The names of the repository/repositories and accession number(s) can be found in the article/supplementary material.
